# Peers are as persuasive as experts in reducing willingness to pay for sugary foods

**DOI:** 10.3389/fnut.2025.1692804

**Published:** 2025-12-02

**Authors:** Nina Arzumanyan, Anna N. Shestakova, Victoria Moiseeva, Isak B. Blank, Vasily Klucharev, Anna Davidovich, Ioannis Ntoumanis

**Affiliations:** 1 International Laboratory of Social Neurobiology, Institute for Cognitive Neuroscience, National Research University Higher School of Economics, Moscow, Russia; 2 Centre for Cognition and Decision Making, Institute for Cognitive Neuroscience, National Research University Higher School of Economics, Moscow, Russia

**Keywords:** healthy eating intervention, social norms, sugar consumption, willingness to pay, peer persuasion, expert persuasion

## Abstract

**Introduction:**

Non-communicable diseases are influenced by multiple genetic, physiological, environmental, and behavioral factors, with dietary sugar consumption representing one of the key modifiable risk determinants. Interventions aimed at reducing sugar intake often rely on persuasive health messaging by experts, yet it remains unclear whether the expertise of the narrator of the message is an indispensable component of a persuasive healthy eating call. To address this question, the present study directly compares the effect of different types of social endorsers on willingness to pay (WTP) for sugary food, including experts and peer endorsers.

**Methods:**

Eighty-eight healthy participants were randomly assigned to three groups: expert (*N* = 30), peer (*N* = 29), and multiple peers (*N* = 29). They evaluated their WTP for sugar-containing, sugar-free, and non-edible products before and after exposure to audio healthy eating interventions delivered by either a nutrition expert or university student peer(s).

**Results:**

All audio interventions significantly decreased participants’ WTP for sugar-containing products compared to sugar-free products. No significant differences were found between the effectiveness of peer and expert persuasion in reducing WTP for sugar-containing products.

**Conclusion:**

Peer-delivered healthy eating calls can be as effective as expert-delivered interventions in decreasing WTP for sugar-containing products. These findings highlight the potential of peer social influence in public health interventions in addition to expert calls.

## 1 Introduction

Excessive sugar consumption has emerged as a significant public health concern, contributing to obesity (1, 2) and non-communicable diseases (3). With global consumption rising steadily over recent decades (4), it is important to understand how individuals can be supported in consuming less sugar. Our daily food choices are embedded within a complex web of social influences that unconsciously shape our dietary patterns, creating an environment where nutritional choices are increasingly structured by external social agents (5–7). Our past research has consistently demonstrated that a healthy eating call endorsed by an expert can decrease consumers’ willingness to pay (WTP) for sugary food (8–10). However, it remains unclear whether the expertise of the narrator of the message is an indispensable component of a persuasive healthy eating call. To address this question, the present study directly compares the effect of different types of social endorsers on WTP for sugary food, including experts and peer endorsers.

Expert endorsers derive persuasive power from perceived competence and trustworthiness (11), with credibility serving as a peripheral cue (12) that influences message acceptance. Peer endorsers, however, operate through identification and internalization processes (13), where individuals adopt attitudes to maintain satisfying relationships with referent groups or because the induced behavior aligns with their value systems. These theories predict distinct persuasion mechanisms: experts should influence through authority-based credibility, while peers should operate through social identification (14) and normative pressure. The critical question is whether these different psychological pathways ultimately converge on the same behavioral outcome, such as reduced valuation of unhealthy food, by successfully altering the perceived value of products.

To examine these distinct persuasion mechanisms, WTP provides a behavioral economics approach that captures how persuasive messages translate into economic valuations. Unlike attitudinal measures, WTP reflects concrete behavioral intentions that more closely predict actual purchase behavior (15). This metric requires participants to make consequential trade-offs between health considerations and monetary costs, reducing hypothetical bias common in stated preference measures where participants tend to overstate their WTP (16–18). Prior WTP research on sugar consumption has focused primarily on visibility enhancements (19) and labeling interventions (20–22), yielding inconsistent results regarding consumers’ valuation of reduced-sugar and sugar-free products (20, 22, 23). However, these approaches represent less influential nudge types (24). The application of WTP to healthy eating calls, which are among the most influential interventions (24) for food nudging, remains underexplored in sugar-related contexts (25). We address this gap by examining WTP for both sugar-containing and sugar-free products, which allows assessment of whether expert credibility and peer identification shift the economic value consumers assign to products.

Expert endorsers have demonstrated effectiveness in shaping food-related purchase behavior by affecting consumers’ WTP. Van Loo et al. (26) showed that expert endorsement, such as the United States Department of Agriculture (USDA) certification, boosts consumers’ WTP for organic chicken, suggesting that trusted expert backing strongly influences purchase decisions. Consistent with this literature, Ntoumanis et al. (8–10) showed that credentialed experts can reduce people’s WTP for unhealthy food. This effectiveness aligns with match-up theory, which suggests that persuasive impact depends on congruence between endorser credentials and message content (27–29). In this case, health experts advocate for health-related behavior change.

However, expertise is not the only pathway to influence. Non-expert endorsers, including celebrities and peers, have also demonstrated significant influence on food choices. Research on peer influence has primarily focused on children and adolescents (30–33) or on other food categories rather than sugar-containing products (34–36). In adult populations, Zhou and Kraak (37) found that millennials are more likely to purchase energy-dense, nutrient-poor products when promoted by celebrities. Peer influence research on adults further demonstrates that food choices are shaped by perceived social norms (38, 39), even in private settings (39), and that healthy eating messages are more persuasive when delivered by peers (40), with individuals being more likely to adopt healthier diets when encouraged by members of their referent group. However, the specific role of peer endorsers in adult sugar consumption remains unexplored.

Direct comparisons of expert versus peer persuasion effectiveness remain limited, particularly for economic valuations for sugar-containing products in adults. Friedman and Friedman (41) demonstrated that endorser-product relevance affects advertising success, while Binder et al. (42) found that only experts’ endorsements affected children’s food selections. However, these studies examined choice behavior rather than WTP, and adult populations, who are more developed autonomy from authority and may respond differently to peer influence than children do. Determining whether expertise is necessary for reducing sugar-containing products valuations could provide valuable insights for designing targeted public health interventions and determining whether expertise itself is the critical factor or if other endorser characteristics might yield similar effects on WTP for food products.

Building on these theoretical mechanisms, our intervention employs affectively-oriented healthy eating calls designed to leverage social influence on emotional food evaluations (24). Since emotions significantly impact food selection (24, 43, 44), and social information can alter affective food evaluations (45), we expect that both expert and peer endorsers may achieve comparable effects by negatively altering liking evaluations of unhealthy foods (42, 45) by mitigating the varying persuasive effects of different endorsement types. This approach aligns with research showing that persuasive messages incorporating firm conclusions, multiple perspectives, and clear reasoning can effectively shape food attitudes (14, 46), particularly when they activate emotional responses that motivate behavior change (47). Our experimental design incorporated both single and multiple peer endorsers, as previous research suggests that multiple social sources can enhance persuasion effectiveness by creating a social consensus effect that strengthens normative influence (12, 48). The multiple peers endorsement also provides greater ecological validity by reflecting real-world social environments where individuals are typically exposed to diverse perspectives within their peer groups, creating a naturalistic representation of opinion formation processes that occur in group settings.

Despite extensive research on persuasion strategies in health communication, Alsubhi et al. (25) identified a critical gap in that no experimental studies have specifically examined consumer WTP for beverages and foods with reduced sugar content. The present study addresses this gap by investigating the comparative effectiveness of expert versus peer persuasion in influencing food choices, more specifically, WTP for sugar-containing and sugar-free products. Based on the literature reviewed above, we hypothesized that peer persuasion would exert a significant impact on decreasing WTP for sugar-containing products (H1), as identification with referent groups can alter product valuation. Furthermore, peer and expert persuasion would demonstrate comparable effectiveness in influencing consumer valuation of these products (H2), as both credibility-based and identification-based persuasion have shown efficacy in shaping food-related attitudes and behaviors. This research contributes to the field by examining whether expert and peer endorsements can effectively shift consumer WTP specifically for sugar-containing products, addressing a methodological gap in healthy eating interventions that have primarily focused on attitudinal outcomes rather than economic valuations.

## 2 Materials and methods

### 2.1 Participants

Ninety healthy adults were recruited to participate in the experiment via online advertisement. Two participants were excluded due to non-compliance with experimental protocols (one statistical outlier and one non-respondent), yielding a final sample of 88 participants. The participants were randomly divided into three distinct groups. During the experiment, the “expert” group (*N* = 30, 18 females, mean age = 21.63 years) listened to a first-person lecture by a nutrition expert, the “peer” group (*N* = 29, 14 females, mean age = 21.41 years) listened to a first-person podcast by a university student, and “multiple peers” group (*N* = 29, 15 females, mean age = 22.21 years) listened to a trialogue between three peers (“multiple peers” group). Inclusion criteria followed Ntoumanis et al. (10): age 18–40 years, right-handed, healthy, normal/corrected vision, no psychiatric/neurological diseases or psychotropic drugs. We excluded participants with eating disorders, metabolic diseases, or those eating sweets less than weekly, as they might lack interest in purchasing sugary products. The participants were asked not to eat for 3 h before the experiment. Supplementary Table 1 summarizes participants’ demographics and eating habits.

The sample size was determined based on a power analysis using G*Power. Given that our main statistical test was a mixed ANOVA examining the interaction between experimental groups and product categories, and given that previous research has identified a relatively small effect size of healthy eating calls on WTP [e.g., (8)], the power analysis was performed with the following parameters: *α* = 0.05, power = 0.9, number of groups = 3, number of measurements = 3, and effect size *f* = 0.18. The statistical power analysis revealed that a sample size of 84 participants would be adequate.

### 2.2 Procedure

**Figure 1:**
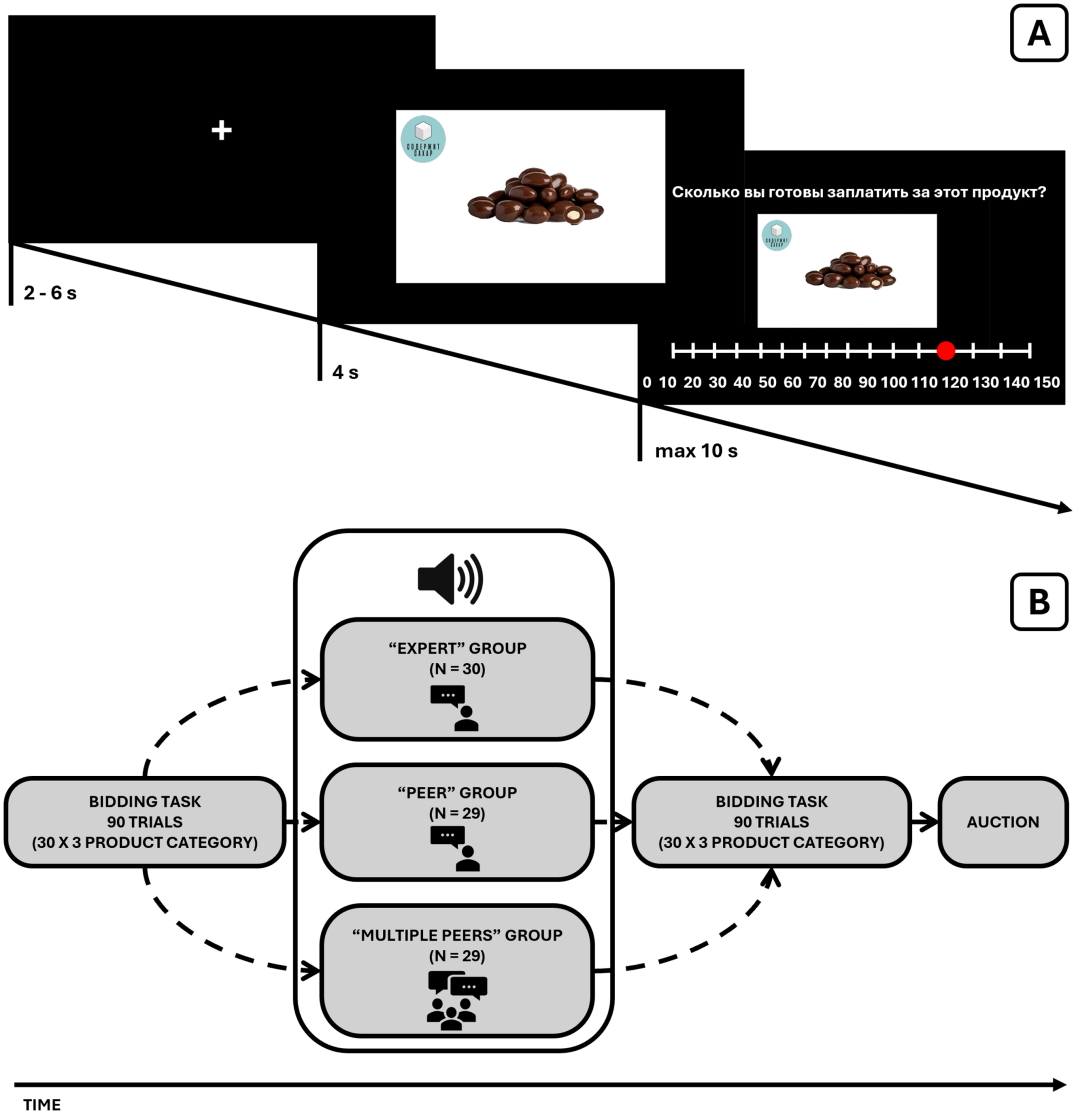
**(A)** Schematic representation of the experimental paradigm. **(B)** The procedure of one of the trials in the bidding task. Each trial followed a three-stage sequence: First, a fixation cross appeared for 2–6 s before each trial. Second, participants viewed a food product for 4 s. Finally, they saw the question “How much are you willing to pay for this product?” and had 10 s to indicate their WTP using a discrete slider ranging from 0 to 150 monetary units (MU) in 10 MU increments.

A schematic representation of the experiment is outlined in Figure 1A. The experiment was conducted in behavioral laboratories located on the university campus. To ensure participants’ attention to the audio messages, each participant was tested individually in a dark, sound-attenuated behavioral cabin that was empty except for the computer screen displaying the experiment. Participants wore headphones throughout the experiment, minimizing distractions and ensuring clear audio delivery. Participants completed an online demographic questionnaire several days prior (see the Questionnaires section) and were informed that our research investigated food preferences. Before the beginning of the experiment, each participant was automatically credited with 150 monetary units as a participation incentive. An additional 150 monetary units were provided for use in the bidding task (see below). Importantly, prior to starting the experiment, we demonstrated to the participants that the products depicted in the images of the bidding task were available and maintained in stock. Participants were thoroughly briefed on the procedure, including that at the end of the experiment, they could acquire one of the previously seen products that was randomly selected by the computer. Each participant completed the experiment in 40 min, including seven practice trials. The practice trials used products selected from the main experimental stimuli, as in Ntoumanis et al. (8–10).

### 2.3 Bidding task

Figure 1B illustrates the structure of one bidding trial. Each stimulus was displayed to the participant for 4 s, followed by the evaluation phase, wherein participants determined their preferred price for the product. Pricing decisions were made using a scale ranging from 0 to 150 monetary units with increments of 10 (e.g., 0, 10, …, 150). If a participant failed to set a price within 10 s, the price of 0 monetary units was automatically assigned.

The bidding task employed the Becker-DeGroot-Marschak auction mechanism (49), where participants could acquire a randomly selected product provided their bid for this product was equal to or exceeded their randomly drawn price from the lottery drum during the auction stage; otherwise, they received nothing and paid nothing. There were two blocks of the bidding task, each consisting of 90 trials, with the product images being identical but presented to participants in a randomized sequence.

Given this auction structure, participants were advised that the optimal strategy for the bidding task was to determine prices based on participants’ current need to acquire the product post-experiment and were instructed to consider having 150 monetary units available for each product.

### 2.4 Stimuli

Ninety full-colored images of products were displayed during the experiment, encompassing three product categories: sugar-containing, sugar-free, and non-edible. Non-edible products served as control stimuli, under the assumption that the healthy eating narrative would not influence participants’ WTP for products outside the targeted categories (9).

All featured products were commercially available, yet product packaging was intentionally omitted to mitigate familiarity bias (50). Products had distinct colored labels: pink, blue, or yellow. Label colors for sugar-containing (pink/blue) and sugar-free (blue/pink) products were counterbalanced between participant groups to control for color effects on nutrition perception (51, 52). Control products consistently had yellow labels.

### 2.5 Persuasive messages

*First-person narration.* The objective of this research was to determine which social endorser—peer or expert—is more effective in promoting healthy eating behaviors. To minimize potential confounding factors influencing the narrative’s indirect effects, the monolog structure of the audio stimuli remained consistent across both single-narrator conditions. Variations were confined to the language used—formal or informal—and the photographs of the narrator displayed to participants.

At the onset of each recording, narrators explicitly stated their social role. In the “expert” group, the role was that of a dietary specialist, specifically a nutritionist (the full English translation of this healthy eating call can be found in Supplementary data 1), while in the “peer” group, it was a university student who hosts a podcast (Supplementary data 2). Both audio recordings presented the same 13 arguments concerning the negative consequences of excessive sugar consumption. The duration of the audio recordings was nearly identical, each lasting approximately 7 min.

The audio for the “expert” group was recorded by a professional male narrator whose effectiveness in persuasive messaging had been validated in prior studies (8–10). To ensure consistency in delivery and minimize variance attributable to vocal characteristics, the same narrator was recorded for the “peer” group. Differences in voice and accent have been proven to impact the persuasiveness of a narrator (53–55). The visual representation of the narrator was consistent across both groups, with only age and attire being adjusted using Photoshop and artificial intelligence tools to suit the respective social roles.

*Third-person narration: Healthy food intervention with multiple social endorsers.* To create a more naturalistic setting for the peer persuasion, an alternative healthy eating call scenario was developed. While the arguments remained unchanged and the informational content was consistent, the structure of this intervention differed notably from the previous two conditions. Firstly, the narrative was restructured into a trialogue format featuring three individuals with different initial perspectives regarding sugar consumption: a female character who was health-conscious and anti-sugar, a male character who was neutral and curious, and another male character who initially defended sugar consumption, but then displayed a shift in perspective (Supplementary data 3). This setup enhanced authenticity by allowing listeners to identify with characters who share their starting viewpoint, with the character’s opinion evolution from pro-sugar to neutrally negative designed to encourage listeners to reconsider their own views and potentially reduce their WTP for sugar-containing products while increasing their WTP for sugar-free alternatives.

The narrators for the healthy eating audio intervention were selected through a voice pretesting with 28 participants (14 females, mean age = 22.75, standard deviation = 2.95) who evaluated 11 voice actors on seven voice characteristics that are usually tested for usage in navigation systems—clear, distracting, trustworthy, assertive, friendly, annoying, entertaining (56). After the ratings were formulated on a seven-point Likert scale (Supplementary Table 2), three voices were selected: one female voice, “f_1,” that significantly outperformed other female candidates, and two male voices, “m_4” and “m_5,” that scored notably higher on “friendly” and “entertaining” dimensions compared to the voice actor, which was recorded in the first-person audio intervention—“m_2.”

### 2.6 Questionnaires

Participants completed four questionnaires at least 2 days prior to the experimental session to assess individual differences that might affect responses to social persuasion. The initial questionnaire gathered demographic information, including gender, age, and education level. Three standardized psychological measures were also included: the Conformity Scale (57, 58) (Cronbach’s alpha *α* = 0.675 in our study), the Consumer Susceptibility to Interpersonal Influence Scale [(59); α = 0.803], and the Big Five Personality traits questionnaire [(60); α = 0.88]. These questionnaires were included since previous studies have reported an association between these personality traits and both sugar consumption and effectiveness of persuasive interventions (61–63).

### 2.7 Data analysis

Statistical analyses were selected in accordance with the research hypotheses. To examine H1 (peer persuasion decreases WTP for sugar-containing food), the data were examined for normality either by using the Shapiro–Wilk test [for data points less than 50; (64)] or quantile-quantile (Q-Q) plots for larger datasets. For normally distributed data, a one-way repeated-measures Analysis of Variance (ANOVA) was implemented, where product category served as a within-subject factor and ΔWTP as the dependent variable. For groups where data were not normally distributed, the Friedman test was conducted as a non-parametric alternative, followed by post-hoc pairwise comparisons using Wilcoxon signed-rank tests. For H2 (comparative efficacy of peer versus expert persuasion), a non-parametric two-way mixed ANOVA [nparLD package in R (65)] was conducted, with the group as a between-subject factor, product category as a within-subject factor, and ΔWTP as the dependent variable. Statistical significance was determined following Benjamini-Hochberg false discovery rate (FDR) correction (66) for multiple comparisons (adjusted *p* < 0.05), consistent with previous research (8–10). Effect sizes were calculated as generalized eta-squared (η^2^) for ANOVA analyses and Kendall’s W for Friedman tests. As for pairwise comparisons between conditions, Cohen’s d was calculated with values of 0.2, 0.5, and 0.8, representing small, medium, and large effects, respectively (67).

The WTP measurement involved calculating mean prices within each of the three product categories (sugar-free, sugar-containing, non-edible; 30 per product category) for each participant. WTP was assessed at pre-intervention (WTP1) and post-intervention (WTP2), with intervention effects quantified as ΔWTP = WTP2—WTP1. The analysis was conducted in RStudio (R version 4.3.1).

To investigate the relationship between participant characteristics measured through questionnaires and effectiveness of persuasion, we used multiple correlation analyses. We assessed whether changes in participants’ WTP across different conditions showed significant correlations with their personality traits. Due to questionnaire scores following a non-normal distribution, we employed Spearman’s correlation coefficient for this assessment.

## 3 Results

### 3.1 Descriptive statistics

Participants submitted bids with the median of 40 MU (IQR = 20–70 MU, mean = 48.15, sd = 38.27). The majority of responses (83.22%) included non-zero bids as in previous studies (15). Response time data revealed an average decision time of 2.78 s (sd = 1.53), with a 0.59% non-response rate.

### 3.2 Peers decrease individuals’ WTP for sugar-containing products

A Friedman test revealed that the “peer” group’s narrative significantly decreased participants’ WTP for sugar-containing products (χ^2^ (2) = 6.81, *p*-value = 0.033, effect size Kendall’s W = 0.117). Post-hoc pairwise comparisons using Wilcoxon signed-rank tests with Benjamini-Hochberg correction confirmed that sugar-containing products had significantly lower ΔWTP compared to both sugar-free products (BH corrected *p*-value = 0.006) and non-edible products (BH corrected *p*-value = 0.029). The difference between sugar-free and non-edible products was not statistically significant (BH corrected *p*-value = 0.213). Since two outliers were identified in this group (WTP < Q1–1.5 × IQR, where Q1 denotes the first quartile and IQR denotes the interquartile range), we repeated the analysis after removing these two participants to verify the robustness of our findings. The removal of these outliers resulted in the data meeting normality assumptions (Shapiro–Wilk test: all *p*-values > 0.05), and thus parametric repeated-measures ANOVA and post-hoc t-tests were conducted. This analysis replicated our findings that there was a significant effect of product category on the ΔWTP (F (2, 52) = 5.95, *p* = 0.005, η^2^ = 0.118) and that the ΔWTP was significantly lower for sugar-containing products than the ΔWTP sugar-free products (t (26) = − 3.32, BH corrected *p*-value = 0.008, Cohen’s d = − 0.84).

The “multiple peers” group demonstrated similar results. A one-way repeated-measures ANOVA yielded a significant effect of the product category (F (2, 56) = 4.95, *p* = 0.01, η^2^ = 0.095). Participants in this condition also displayed significant decrease in WTP for sugar-containing products compared to the ΔWTP for sugar-free products (t (28) = − 2.59, BH corrected *p*-value = 0.023, Cohen’s d = − 0.68) and for non-edible products (t (28) = − 2.61, BH corrected *p*-value = 0.023, Cohen’s d = − 0.64). No significant difference was observed between the ΔWTP for sugar-free and non-edible products (t (28) = − 0.563, BH corrected *p*-value = 0.578, Cohen’s d = 0.13).

Supplementary Table 3 provides a comprehensive overview of Spearman correlation coefficients between participants’ age and personality trait scores and ΔWTP across different product categories. The Spearman correlation analysis revealed that age was negatively correlated with WTP for sugar-containing products (rho = −0.243, *p* = 0.0228). Similarly, agreeableness also exhibited a significant negative correlation (rho = −0.227, *p* = 0.0331) with WTP for sugar-containing products.

### 3.3 Experts also decrease individuals’ WTP but show no advantage over peers

Similar to our previous studies (8–10), we found a significant effect of the product category in the “expert” group (χ^2^ (2) = 6.62, *p*-value = 0.0366, effect size Kendall’s W = 0.11). Participants significantly decreased their WTP for sugar-containing products compared to sugar-free products (BH corrected *p*-value = 0.02).

**Figure 2:**
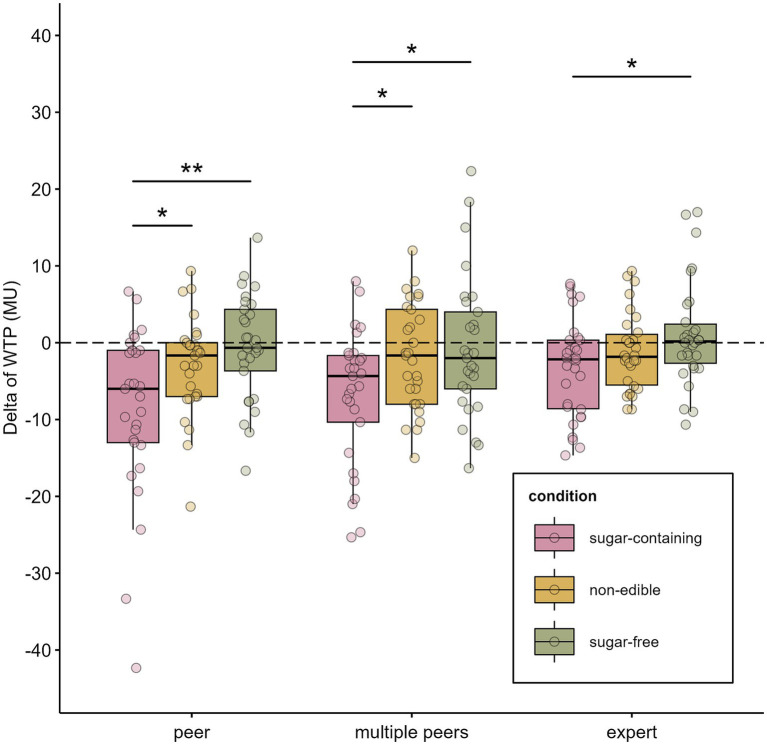
The comparison of the effects of peer and expert persuasion on the WTP for sugar-containing, non-edible and sugar-free products. The ΔWTP for different categories of products—sugar-containing, non-edible and sugar-free. Each dot represents the ΔWTP of a single participant. Statistically significant differences between conditions are denoted with asterisks (*p-value < 0.05, **p-value < 0.01).

A non-parametric two-way mixed ANOVA comparing all three social endorser groups (Figure 2) revealed no statistically significant difference between groups (*F* (1.98) = 1.36, *p*-value = 0.256, η^2^ = 0.026). There was no significant interaction between group and the product category (*F* (3.91) = 0.47, *p*-value = 0.754, η^2^ = 0.010), indicating that the pattern of willingness to pay for different product categories was consistent regardless of endorser type. Follow-up pairwise comparisons between groups provided additional detail about specific group contrasts. When directly comparing “peer” and “expert” groups, no significant interaction between group and the product category (*F* (2, 114) = 1.52, *p*-value = 0.222, η^2^ = 0.014) was observed. This finding was replicated between “multiple peers” and “expert” groups with the second comparison yielding similar results (F (2, 114) = 0.73, *p*-value = 0.485, η^2^ = 0.007).

In addition, a sensitivity analysis excluding two extreme outliers from the peer persuasion group (*n* = 27, for “peer” group after removal) replicated the original findings. A non-parametric two-way mixed ANOVA revealed no statistically significant difference between groups (*F* (1.99) = 1.17, *p*-value = 0.309, η^2^ = 0.026) and no significant interaction between group and the product category (*F* (3.9) = 0.41, *p*-value = 0.794, η^2^ = 0.010), further verifying that the pattern of ΔWTP was consistent regardless of endorser type.

## 4 Discussion

This study explored the effects of peer persuasion on people’s WTP for sugar-containing and sugar-free products. Healthy eating calls presented by experts and by peers lacking expertise regarding the negative consequences of excessive sugar consumption, both significantly decreased participants’ WTP for sugar-containing products.

Importantly, WTP for sugar-containing products significantly decreased across all endorsers of healthy eating calls—“expert,” “peer” and “multiple peers” audio interventions. Our results are consistent with our hypothesis H1 and with findings from Robinson and Higgs (45), who suggested that negative social information about the products more frequently leads to the rejection of unhealthy alternatives as opposed to the acceptance of new healthier options. Notably, peer-delivered interventions produced a similar decrease in WTP as expert-delivered interventions for sugar-containing products. Although it aligns with previous studies (5, 68, 69) showing that peer persuasion can effectively change food choices, our research shows that it also lowers purchase intentions and WTP for sugar-containing products. It does not generally imply that two persuasive powers are equal; rather, peer and expert endorsements are viewed similarly for WTP valuations in sugar consumption.

We hypothesized that peer persuasion would be as effective as expert persuasion for two key reasons. First, social norms conveyed by members of an individual’s referent group can be interpreted as implicit social evaluation, with deviations discouraged through social approval or disapproval (13, 40). Second, because food products involve relatively low purchase risk, consumers may be receptive to influence from similar consumers who share comparable product experiences and have acquired practical knowledge through actual consumption (41). Consistent with our expectations, peer persuasion was equally as effective as expert persuasion in our study. This finding suggests that experiential expertise gained through personal consumption may be as persuasive as formal professional expertise in healthy eating interventions, challenging traditional assumptions about the superiority of credentialed endorsers in low-risk consumption contexts (11, 70, 71).

Our results suggest peer endorsers effectively shift food preferences and choices, contrasting with Binder et al. (42), who found that only expert endorsers significantly influenced children’s food choices, while peer and celebrity endorsers did not produce significant effects. Our findings suggest peer endorsers can effectively reduce consumers’ WTP for sugar-containing products and raise awareness about excessive sugar consumption consequences. This difference in findings regarding peer influence may be attributed to differences in target populations, contextual factors, or the specific nature of the messages being communicated about sugar consumption versus general healthy eating.

Importantly, socio-demographic factors such as gender, age, BMI index, income and education have proven to affect the WTP of customers for the healthier food options (36, 72). We did not find proof for this in our previous studies regarding expert persuasion in relation to food preferences (8, 9). However, our study suggests that agreeableness and age exerted an effect on the ΔWTP, suggesting that both older individuals and those scoring higher on agreeableness tend to value less sugar-containing products and bid less after listening to the persuasive message. However, these findings have to be considered with caution due to the weak magnitude of the correlation coefficients. In addition, since the age range of our participants was limited, these results cannot be generalized to other age groups, and generational differences in responsiveness to health messaging are likely.

The absence of differential effects between social endorser types may be attributed to familiarity bias surrounding healthy eating messages, as we discussed above. Public health information about sugar consumption is ubiquitous across various channels (73), potentially saturating persuadees with prior knowledge from both expert and peer sources. In their research on message familiarity, Claypool et al. (74) demonstrated that repeated exposure to health information can lead to processing fluency that overshadows source characteristics in persuasion contexts. To address this limitation, future research concerning the neurocognitive mechanism of persuasion in relation to healthier food choices could investigate topics with lower public awareness while maintaining relevance to nutrition. The choice might fall on lesser-known nutritional compounds (75) or employing novel yet plausible nutrition claims where source credibility might play a more decisive role in persuasion outcomes (76). Such approaches would more effectively isolate the impact of endorser type on message persuasiveness by reducing the confounding effect of prior knowledge.

While our methodology employed audio narratives to simulate social interactions, questions regarding ecological validity remain. Although these narratives provide a more controlled experimental approach than purely text-based stimuli, they may not fully capture the complexity of real-world endorsements where multiple channels of communication operate simultaneously (77). As Schmuckler (78) noted, increasing participants’ immersion in social scenarios often yields behavior more predictive of real-life responses, suggesting that future studies might benefit from more interactive paradigms. Additionally, Wilson et al. (79) demonstrated that laboratory studies with enhanced ecological validity yield stronger correlations with field observations. Our study aimed to strike a balance between experimental control and naturalistic context; however, incorporating more dynamic interaction could strengthen future investigations in this domain. Finally, our results should be replicated using different types of narratives and in clinical populations, as this study included only healthy participants and employed only three types of narratives, which may limit the generalizability of findings.

Related to this balance between experimental control and real-world authenticity, participants made real choices with the understanding that they would receive a product at the end of the experiment. This design is intended to elicit genuine WTP decisions (15), yet they used money provided for the experiment rather than their own. Although Plassmann et al. (15) demonstrated that WTP measures using this incentive-compatible method are reliable predictors of value, it still may differ from actual monetary transactions where participants experience direct financial loss.

In conclusion, we found that peer-delivered healthy eating calls can effectively decrease WTP for sugar-containing products. Our findings demonstrate that peers can successfully influence consumers’ purchase intentions, with no significant difference in effectiveness between peer and expert endorsers in reducing WTP for sugar-containing products. Importantly, our study provides evidence that the credentialed expertise of the persuader may not be indispensable for influencing WTP for healthier food options.

## Data Availability

TThe datasets presented in this study can be found in online repositories. The names of the repository/repositories and accession number(s) can be found online at https://osf.io/zqaen/, and in the article - Supplementary material.
