# Supplementary material for: Peers are as persuasive as experts in reducing willingness to pay for sugary foods
Source: Front Nutr. 2025 Dec 2;12:1692804. doi: 10.3389/fnut.2025.1692804 (PMC12705371; doi:10.3389/fnut.2025.1692804)
Supplement: Supplementary file 1 [file Data_Sheet_1.PDF]

Supplementary Material

Supplementary Tables

Supplementary Table 1. Sociodemographic characteristics of participants by intervention group. Baseline demographic characteristics of participants allocated to “expert” (n = 30), “peer” (n = 29), and “multiple peers” (n = 29) groups. Values represent frequency (n) and percentage (%) for each characteristic within each group, with full sample totals provided in the final columns. Percentages are rounded to whole number.

	**“expert” group**		**“peer” group**		**“multiple peers” group**		**full sample**	
	n	%	n	%	n	%	n	%
**gender**								
female	18	60	14	48	15	52	47	60
male	12	40	15	52	14	48	41	40
**age**								
18 – 23 years old	23	77	26	90	23	79	72	82
> 23 years old	7	23	3	10	6	21	16	18
**BMI**								
under	4	13	3	10	1	3	8	9
normal	22	73	20	69	23	79	65	74
over	4	13	6	21	5	17	15	17

**highest education level**								
no education	0	0	0	0	0	0	0	0
incomplete secondary education	0	0	0	0	0	0	0	0
complete secondary education	7	23	13	45	4	14	24	27
incomplete secondary specialized education	2	7	0	0	0	0	2	2
secondary specialized education	0	0	1	3	1	3	2	2
incomplete higher education	8	27	4	14	10	35	22	25
higher education	13	43	11	38	14	48	38	43
academic degree (PhD equivalent)	0	0	0	0	0	0	0	0
**sugar consumption habits**								
no sugar	0	0	0	0	0	0	0	0
1 or few times a month	2	7	0	0	2	7	4	5
once a week	3	10	4	14	0	0	7	8
few times a week	8	27	12	41	9	31	29	33
once a day	10	33	7	24	10	35	27	31
twice a day	6	20	4	14	5	17	15	17
3 or more times a day	1	3	2	7	3	10	6	7

Supplementary Table 2. Overall likeability and voice characteristics (annoying, assertive, clear, distracting, entertaining, friendly, trustworthy) of narrator voices rated on a scale from 1 to 7. Mean values and standard deviations are presented for each characteristic. The notation "f" and "m" denote female and male voice actors respectively, with numbers 1-8 serving as identification codes.

**actor_id**		**f_1***	**f_2**	**f_3**	**m_1**	**m_2****	**m_3**	**m_4***	**m_5***	**m_6**	**m_7**	**m_8**
**voice_feature**												
overall	**mean**	**5.25**	**3.93**	**3.43**	**3.64**	**4.46**	**5.04**	**5.32**	**5.04**	**3.82**	**4.82**	**4.39**
likeability	sd	1.29	1.33	1.62	1.62	1.62	1.75	1.09	1.45	1.49	1.28	1.57
annoying	**mean**	**2.43**	**3.89**	**4.96**	**4.32**	**3.89**	**2.46**	**2.64**	**2.75**	**4.25**	**3.18**	**3.14**
	sd	1.26	1.91	1.79	1.61	2.1	1.69	1.45	1.67	1.92	1.66	1.48
assertive	**mean**	**5.39**	**4.21**	**4.82**	**4.25**	**5.11**	**5.71**	**5.21**	**5.29**	**4.54**	**4.82**	**5.43**
	sd	1.34	1.34	1.22	1.38	1.57	1.33	1.29	1.3	1.67	1.39	1.03
clear	**mean**	**5.79**	**5.46**	**5.5**	**5.29**	**5.64**	**6.04**	**6.07**	**6.04**	**5.5**	**5.82**	**6**
	sd	1.5	1.32	1.43	1.27	1.25	1.32	0.9	1.1	1.32	1.06	1.19
distracting	**mean**	**2.25**	**3.93**	**4.86**	**4.04**	**3.79**	**2.57**	**2.71**	**2.68**	**3.82**	**2.68**	**2.75**
	sd	1.35	1.7	1.98	1.84	1.83	1.55	1.3	1.22	1.72	1.42	1.46
entertaining	**mean**	**4.89**	**3.39**	**3.61**	**3.5**	**3.86**	**4.32**	**4.96**	**4.82**	**3.89**	**4.43**	**4.21**
	sd	1.37	1.62	1.4	1.55	1.8	1.66	0.92	1.66	1.62	1.32	1.45
friendly	**mean**	**4.71**	**3.89**	**4.11**	**4.11**	**4.18**	**4.43**	**5.11**	**4.64**	**4.82**	**4.82**	**4**
	sd	1.27	1.45	1.42	1.1	1.36	1.4	0.99	1.13	1.54	1.22	1.54
trustworthy	**mean**	**5.04**	**3.75**	**3.71**	**3.75**	**4.5**	**5.21**	**5**	**5.18**	**3.82**	**4.39**	**4.89**
	sd	1.29	1.04	1.46	1.29	1.55	1.6	1.05	1.31	1.47	1.52	1.5

* Voices selected for the “multiple peers” group audio intervention recording.

** Voice used in single-narrator audio interventions - “expert” and “peer” groups.

Supplementary Table 3. Spearman Correlation Coefficients (ρ) between personality traits and ΔWTP. The associations between personality traits and ΔWTP for sugar-containing, non-edible and sugar-free products following exposure to healthy eating audio interventions. Values represent correlation coefficients between individual personality characteristics and ΔWTP. *p < 0.05

	**ΔWTP**		
	sugar	non-edible	sugar-free
**personality variables**			
age	-0.243*	0.009	-0.067
social conformity	-0.061	-0.099	0.012
consumer susceptibility to interpersonal influence	-0.038	0.047	0.130
extraversion	-0.154	-0.070	-0.018
agreeableness	-0.227*	-0.145	-0.040
conscientiousness	0.169	-0.119	0.034
neuroticism	-0.011	0.041	0.022
openness	-0.014	-0.147	-0.098

Supplementary Data

Supplementary Data 1. English translation of the audio-delivered healthy eating intervention script for the "expert" group. The intervention is presented in a form of a lecture delivered by a doctor.

Hello, my name is Andrei Butlerov, I'm a medical doctor in the Clinic of the Research Institute of Nutrition and I teach Nutrition at the first medical university in Moscow.

Let me start with a question: What would happen if you stopped eating sugar? Perhaps your life wouldn't be very "sweet" anymore. Seriously speaking, eating too much sugar can seriously affect your health. It leads to weight gain, sharp mood swings and even addiction. Some of my nutritionist colleagues consider sugar to be even more dangerous than fat, but we still don't pay enough attention to the amount of sugar we consume every day. In today's lecture, I'm going to reveal what would happen to your heart, skin and brain if you quit sugar – and even if it's worth the effort. I will give you thirteen important arguments about sugar consumption.

Argument 1. You will overcome a serious addiction. Studies show that sugar is actually more addictive than cocaine. Lab rats were given cocaine until they became addicted to it. Then, they were given a choice: Either to continue taking cocaine or switch to sugar. Guess what. 94% chose sugar.

Argument 2. Your breath will be better. Sugar provides a source of food for bacteria. In these conditions, bacteria reproduce more quickly, which results in a terrible breath.

Argument 3. You will increase your brain power. Studies have shown that sugar hinders activities, such as learning and memorizing things. So, believe it or not, when preparing for the exam, it's better to stay away from chocolate and other sugar-containing kinds of food.

Argument 4. You will be less likely to have Alzheimer's disease. There is a chemical in your brain, known as BDNF – brain derived neurotrophic factor. It helps the brain to form connections and make new memories. Drop in the factor has been linked to eating excessive amounts of sugar.

Argument 5. Your skin will look younger. A study in the American Journal of Clinical Nutrition suggests that giving up sugar may result in your acne disappearing. Sugar is inflammatory and inflammation is known as a cause of acne.

Argument 6. Your heart will thank you. People who eat too much sugar have a higher risk of having a heart attack. Another important thing is that giving up sugar will lead to the decrease of insulin level and drop of the average heart rate. Your blood pressure will also decrease, which means that your heart and your vessels will have less work to do. So, conditions such as stroke and artery disease will become less likely.

Argument 7. You will have less bad cholesterol. High sugar intake raises levels of bad cholesterol and blood fats that can clog your blood vessels and lead to severe heart conditions.

Argument 8. You will be in a much better mood. Yes, being pretty irritable right after you say "no" to sugar is normal. But! When you're done with your diet change, you will feel much better. Studies have found that people who eat very little sugar or not at all, are rarely diagnosed with depression.

Argument 9. Your day/night cycle will be more naturally balanced. High sugar intake interferes with your normal energy level. It's ok to feel sleepy after giving up sugar, but when your body adapts to it, you'll be very active throughout the day and ready to sleep when bedtime comes. You'll finally get a good sleep.

Argument 10. You will be less likely to have diabetes. When you eat too much glucose, high insulin resistance develops. It means that sugar can't get into your cells and can get stuck in the bloodstream. This leads to pre-diabetes condition and eventually, to the real disease.

Argument 11. Your eyesight will be sharper. Sugar also affects the health of your eyes. Fluctuations of insulin and glucose levels lead to vessel damage and decrease blood supply to your eyes. As a result, the eye sharpness drops and conditions such as myopia and cataract may develop.

Argument 12. You will lose weight fast. When you eat a lot of sugar, especially fructose, you are very likely to overeat. The pancreas is forced to produce more insulin, so other hormones that regulate metabolism, digestion and weight are neglected. So, the body sends you wrong signals that it is time to have a snack. Don't let this cunning sugar fool you.

Argument 13. You will see your doctor less often. Your immune system is seriously affected by consumption of large amounts of sugar. You are protected from bacteria and viruses by white blood cells. Their efficiency drops dramatically when you eat simple sugars, like glucose, fructose and honey. Sugar also causes the development of cavities and gum disease, so you have a chance not only to see your doctor less often, but also your dentist. I don't think you'll be missing them a lot.

That's all about the health benefits of giving up sugar. What about the enjoyment we feel from eating something sweet though? Doesn't sugar make products taste better? Well, I have good news. Sugar-free products can still be tasty, especially when they contain sugar substitutes that provide a similar sweet taste to that of sugar. This is achieved without adding as many calories as regular sugar would.

Supplementary Data 2. English translation of the audio-delivered healthy eating intervention script for the "peer" group. The intervention is presented in a form of a university podcast delivered by a student.

Hi everyone! Welcome to our university podcast! My name is Andrei Butlerov, and today I'm going to tell you about how exactly your heart function, skin condition, and brain will improve if you give up sugar – even though it will require some effort.

Let me start with a question: what will happen if you stop eating sugar? Perhaps your life won't be so "sweet" anymore. But seriously speaking, consuming too much sugar can have a very strong impact on your health. Sugar consumption leads to weight gain, dramatic mood swings, and even addiction. Sugar is even more dangerous than trans fats, yet we still don't pay enough attention to how much sugar we consume every day.

I can't call myself an expert in this field, but I know 13 risks associated with sugar consumption, and today I want to share them with you. So, let's begin!

Argument 1. You will overcome a serious addiction. Studies show that sugar is actually more addictive than cocaine. Lab rats were given cocaine until they became addicted to it. Then, they were given a choice: Either to continue taking cocaine or switch to sugar. Guess what. 94% chose sugar.

Argument 2. Your breath will be better. Sugar provides a source of food for bacteria. In these conditions, bacteria reproduce more quickly, which results in a terrible breath.

Argument 3. You will increase your brain power. Studies have shown that sugar hinders activities, such as learning and memorizing things. So, believe it or not, when preparing for the exam, it's better to stay away from chocolate and other sugar-containing kinds of food.

Argument 4. You will be less likely to have Alzheimer's disease. There is a chemical in your brain, known as BDNF – brain derived neurotrophic factor. It helps the brain to form connections and make new memories. Drop in the factor has been linked to eating excessive amounts of sugar.

Argument 5. Your skin will look younger. A study in the American Journal of Clinical Nutrition suggests that giving up sugar may result in your acne disappearing. Sugar is inflammatory and inflammation is known as a cause of acne.

Argument 6. Your heart will thank you. People who eat too much sugar have a higher risk of having a heart attack. Another important thing is that giving up sugar will lead to the decrease of insulin level and drop of the average heart rate. Your blood pressure will also decrease, which means that your heart and your vessels will have less work to do. So, conditions such as stroke and artery disease will become less likely.

Argument 7. You will have less bad cholesterol. High sugar intake raises levels of bad cholesterol and blood fats that can clog your blood vessels and lead to severe heart conditions.

Argument 8. You will be in a much better mood. Yes, being pretty irritable right after you say “no” to sugar is normal. But! When you’re done with your diet change, you will feel much better. Studies have found that people who eat very little sugar or not at all, are rarely diagnosed with depression.

Argument 9. Your day/night cycle will be more naturally balanced. High sugar intake interferes with your normal energy level. It’s ok to feel sleepy after giving up sugar, but when your body adapts to it, you’ll be very active throughout the day and ready to sleep when bedtime comes. You’ll finally get a good sleep.

Argument 10. You will be less likely to have diabetes. When you eat too much glucose, high insulin resistance develops. It means that sugar can’t get into your cells and can get stuck in the bloodstream. This leads to pre-diabetes condition and eventually, to the real disease.

Argument 11. Your eyesight will be sharper. Sugar also affects the health of your eyes. Fluctuations of insulin and glucose levels lead to vessel damage and decrease blood supply to your eyes. As a result, the eye sharpness drops and conditions such as myopia and cataract may develop.

Argument 12. You will lose weight fast. When you eat a lot of sugar, especially fructose, you are very likely to overeat. The pancreas is forced to produce more insulin, so other hormones that regulate metabolism, digestion and weight are neglected. So, the body sends you wrong signals that it is time to have a snack. Don’t let this cunning sugar fool you.

Argument 13. You will see your doctor less often. Your immune system is seriously affected by consumption of large amounts of sugar. You are protected from bacteria and viruses by white blood cells. Their efficiency drops dramatically when you eat simple sugars, like glucose, fructose and honey. Sugar also causes the development of cavities and gum disease, so you have a chance not only to see your doctor less often, but also your dentist. I don’t think you’ll be missing them a lot.

That’s all about the health benefits of giving up sugar. What about the enjoyment we feel from eating something sweet though? Doesn’t sugar make products taste better? Well, I have good news. Sugar-free products can still be tasty, especially when they contain sugar substitutes that provide a similar sweet taste to that of sugar. This is achieved without adding as many calories as regular sugar would.

That's all for today! Thank you all very much for your attention, you'll hear from me in a week. Have a great week everyone, bye!

Supplementary Data 3. English translation of the audio-delivered healthy eating intervention script for the “multiple peers” group. The intervention is presented in the form of a trialogue between student friends.

Me: Well, three packets for me. Anyone else needs sugar?

Person 1: What? And all these sugar packets are for you only? Three pieces?

Person 2: Let him drink it, however he wants, if he likes it better this way. Don’t make a big deal out of it.

Person 1: Ah yeah? Let's see how much of a deal it will be when his teeth will fall out. Do you want that to happen?

Me: Why are you like this...

Person 1: Because I care about you? That's why.. I understand how difficult it is to reduce sugar consumption because it's a real addiction. I saw a study that showed that sugar was more addictive to the body than cocaine. Lab rats were given cocaine until they became addicted to it. Then, they could choose to either keep taking cocaine or switch to sugar, and most of them chose sugar!

Person 2: Really? That's an interesting fact, by the way, I didn't know that.

Me: Yeah, I've always actually been interested in this topic, I also remember that sugar affects people's moods. And that people who eat a lot of sugar are more prone to depressive moods than people who eat little or no sugar. But I never took these facts seriously.

Person 2: Seriously? But look, you don't always add sugar! We go here quite often, and it seems to me that you're more likely to take more sugar packets when you're tired and you need some extra energy.

Me: Hm, well...let me think. I think I do that, but not on purpose.

Person 1: Yeah, that's understandable, but you realize that, fine, you'll have energy for a while but then you will feel sleepy again.

Person 2: According to my previous observations, if I ate something sweet before going to bed, then I couldn’t fall asleep quickly.

Me: Well, it is always said that sleep patterns can get disrupted a lot by various factors, and sugar is one of them. And once people stop eating sugar and their body adapts to this change, they become very active during the day, and when they have to go to bed, they fall asleep easily.

Person 2: Yeah, well, that makes sense, actually. I've definitely heard that before, too. It's just, you know, sometimes I don't even want to eat, like when I'm nervous about exams or something. And then I just study all day and drink tea or coffee with sugar so I can at least eat something during the day.

Me: Oh, true, I do that a lot, too. It's kind of a habit.

Person 1: Guys! You shouldn't do that at all. I read somewhere that it's better to stay away from chocolate and other sugary foods while studying for exams, but I can't remember why.

Me: No way, interesting, I always thought, on the contrary, that everyone believes that chocolate makes us smarter.

Person 2: No, no, really! I also read about that!

Person 1: They studied the cause and effect of sugar consumption and found out brain performance is decreased by sugary foods. So, studies have shown that if you eat too much sugar, it becomes a little bit more difficult to learn something new, and, even more difficult, to remember it.

Me: Can you send me that article later, please, so I can read it. Really thought it was the other way around.

Person 1: Sure.

Person 2: By the way, I just recently had an unforgettable experience, I was at the doctor's the other week, and my therapist told me, that I should follow a diet for now, because I have an upper limit of blood sugar, she told me a million things about diabetes, cholesterol, and so on. For some reason, she was describing everything very detailly, the only thing I didn't quite understand was why I needed the information, that the risks of developing Alzheimer's disease are significantly reducing by decreasing the glucose level. That there is some kind of chemical in our brain, I guess is known as BDNF - brain neuron repair factor. It helps the brain to form connections and create new memories.

Person 1: I don't really get it, and sugar has some effect on that level?

Person 2: Yes, in other words, the decreased BDNF level is associated with eating too much sugar.

Person 1: Oh, okay. Well, listen, I think you should listen to what you were told by your doctor, you better not mess around with this whole thing.

Person 2: Yes, yes, I really started to get worried, because I'm always very calm about this topic. I also add sugar, but now I'll try to add less, and if it works, after a while I'll stop adding it at all.

Me: The main thing, please, don't worry too much. I am sure that everything will be fine, but for now you should not eat and drink sweet things.

Person 2: Yes, I'm trying, it's just that I also remembered that there's an even greater chance of getting diabetes. When you eat too much glucose, the sugar can't get inside the cells and stays in the bloodstream. And that leads to a pre-diabetic state and eventually to the onset of the disease. We were told this at a lecture the other day.

Person 1: When I imagine that diabetics have to inject themselves with insulin several times a day, I get scared.

Me: Yeah, me too, to be honest.

Person 2: Well, at least she didn't tell me about it. But, of course, there's a million things, not only that, it also has a big effect on the functioning of the heart.

Person 1: Yes! That's what I was going to say!

Person 2: The only thing, I don't remember how, to be honest, but I'll google it and read aloud, I think it will be interesting.

Person 1: Yeah, and while you are googling it, and I'll tell you what I just remembered myself.

Me: * laughter * It doesn't scare you how much we know about it, just because of everyday things. Okay, okay, tell me.

Person 1: * laughter * Yes, true! So, do you know that sugar is a food source for bacteria. In a "sweet" environment, the bacteria develop faster, which leads to bad breath.

Me: No, I didn't know that. Then, I better avoid eating sugar before I see my girlfriend.

Person 2: * laughter * Don't worry, I will also tell her about this fact. Not only you have to suffer.

Me: Yes, true, thank you.

Person 1: Yes, such a strange fact that I somehow remembered it. Well? How is the google search going? Found anything?

Person 2: Yes! People who eat too much sugar are prone to a higher risk of heart attack. Another important thing that is written here is that giving up sugar leads to lower insulin levels.

Me: I don't really understand what you're saying.

Person 2: I'm sorry, I'll interrupt you, I'll finish reading, or I'll lose it later, and then we'll figure it out. It also leads to a decrease in the average heart rate. In this case, the blood pressure also goes down, which means the heart and blood vessels have to work less.

Person 1: There it is! And so, if you don't eat sugar, the risk of conditions like stroke and arterial disease goes down!

Me: Give me the phone, please, I still don't really understand what you are talking about.

Person 2: Yes, of course, I'm sorry, read here.

Me: That's it, got it. Well, yeah, with all those side effects, in fact, it's not worth eating so many sugary sweets. Did you see the next search query here? It is an article about how sugar-containing foods affect vision.

Person 2: No, *laughter*, read it. It seems to me, that we probably learn everything today about sugar and its influence on the body.

Me: "*TOP-5 facts about sugar you never thought about*"... Well, we've already discussed that.

Person 1: Read only new things!

Me: Found it! Sugar has a big effect on the health of your eyes. The fluctuations in insulin and glucose levels lead to damages in the vessels in the retina and decrease in blood flow. As a result, visual acuity worsens, and myopia and cataracts may develop.

Person 1: Well, here's another reason!

Person 2: You know, what I also remembered: my parents always worried a lot about cholesterol. High sugar intake increases the levels of "bad" dangerous cholesterol and fats in the blood, which clog your blood vessels and lead to...

Me: Apparently again, to severe heart diseases.

Person 2: That's right! I think the less you eat and drink sugar-containing products, the less you visit the doctor.

Person 1: Yes, that's right.

Person 2: Because it makes sense that consuming lots of sugar seriously affects our immune system. So if you cut back on your sugar intake, you will have to go to the doctors less often. I don't think we will get too upset about that.

Person 1: Oh, by the way, there's more! I think, you remember, because you knew me when I had serious skin problems. And I read a lot about possible causes of my skin problems, and one of them was sugar consumption!

Person 2: Oh, that's back when you weren't living a healthy lifestyle yet!

Me: I had already forgotten about that period of your life. Now you don't associate with that kind of person at all.

Person 1: Yeah, I don't really want to remember the way I ate back then, neither. It was a nightmare. So, I came across an article in the American Journal of Clinical Nutrition that showed that avoiding sugar can make acne disappear. Sugar is known for causing inflammations, which lead to acne.

Person 2: Yeah, my sister is now fighting acne. The teenage era we all want to forget.

Me: Facts.

Person 1: You told me recently that you were trying to lose weight. You know that when you eat a lot of sugar, especially fructose, you probably overeat.

Person 2: That's true, by the way.

Person 1: There's something about the pancreas, and that it needs to produce more insulin, I guess, ignoring other hormones that regulate metabolism, digestion, and weight. And so, the body starts sending the wrong signals that it's time to snack and that we're hungry.

Me: Yeah, I know about that, but as you can see, it's not working for me yet.

Person 2: I think if you will get over this need for sweets for a little bit, you'll get used to it quickly. At least I don't suffer much now.

Person 1: Well, yeah, especially nowadays they offer sweeteners everywhere, and if it's really hard, you can try this method.

Me: Well, I also thought about it, but for some reason everyone thinks that when the products have real sugar, they taste better.

Person 2: Speaking of which! I recently tried a candy bar with sweeteners, because in the cafe I was told, that they tasted better than regular candy bars for some reason. Decided to try it, and basically, there's no real difference there. So I think sugar-free foods, in general, can be just as tasty!

Me: Wait, I think they have them here too. Okay, I'm going to try them.

Person 1: Well, yes, come on! In this case, you, in fact, follow your diet!

Me: Yes! I just thought about that.
